# Targeting Cancer Associated Fibroblasts in Liver Fibrosis and Liver Cancer Using Nanocarriers

**DOI:** 10.3390/cells9092027

**Published:** 2020-09-03

**Authors:** Leonard Kaps, Detlef Schuppan

**Affiliations:** 1 Institute of Translational Immunology and Research Center for Immune Therapy, University Medical Center, D-55131 Mainz, Germany; leonardkaps@googlemail.com; 2 Deptartment of Medicine 1, University Medical Center, D-55131 Mainz, Germany; 3 Division of Gastroenterology, Beth Israel Deaconess Medical Center, Harvard Medical School, Boston, MA 02115, USA

**Keywords:** nanoparticle, tumor microenvironment (TME), extracellular matrix (ECM), immune suppression, therapy, tumor-associated-macrophage (TAM), anti-fibrotic, siRNA, anti-fibrotic, HCC, desmoplastic stroma, platelet-derived growth factor (PDGF)

## Abstract

Cancer associated fibroblasts (CAF) and the extracellular matrix (ECM) produced by them have been recognized as key players in cancer biology and emerged as important targets for cancer treatment and drug discovery. Apart from their presence in stroma rich tumors, such as biliary, pancreatic and subtypes of hepatocellular cancer (HCC), both CAF and certain ECM components are also present in cancers without an overt intra-tumoral desmoplastic reaction. They support cancer development, growth, metastasis and resistance to chemo- or checkpoint inhibitor therapy by a multitude of mechanisms, including angiogenesis, ECM remodeling and active immunosuppression by secretion of tumor promoting and immune suppressive cytokines, chemokines and growth factors. CAF resemble activated hepatic stellate cells (HSC)/myofibroblasts, expressing α-smooth muscle actin and especially fibroblast activation protein (FAP). Apart from FAP, CAF also upregulate other functional cell surface proteins like platelet-derived growth factor receptor β (PDGFRβ) or the insulin-like growth factor receptor II (IGFRII). Notably, if formulated with adequate size and zeta potential, injected nanoparticles home preferentially to the liver. Several nanoparticular formulations were tested successfully to deliver dugs to activated HSC/myofibroblasts. Thus, surface modified nanocarriers with a cyclic peptide binding to the PDGFRβ or with mannose-6-phosphate binding to the IGFRII, effectively directed drug delivery to activated HSC/CAF in vivo. Even unguided nanohydrogel particles and lipoplexes loaded with siRNA demonstrated a high in vivo uptake and functional siRNA delivery in activated HSC, indicating that liver CAF/HSC are also addressed specifically by well-devised nanocarriers with optimized physicochemical properties. Therefore, CAF have become an attractive target for the development of stroma-based cancer therapies, especially in the liver.

## 1. Introduction

Hepatocellular carcinoma (HCC) is the most frequent form of primary liver cancer (80%), followed by cholangiocellular carcinoma (6%) and other rare types. The overall burden of HCC is increasing, HCC being the second leading cause of life lost from cancer worldwide between 2005 and 2015 and with an increase of 4.6% in years of life lost (95% CI −1.6% to 15.4%) [1]. The majority of HCCs (>80%) develop in cirrhotic livers, suggesting that the altered microenvironment in cirrhosis plays an important role in the development and growth of this epithelial cancer. Cirrhosis, an advanced stage of fibrosis, represent the end-stage of virtually every chronic liver disease. Histologically, cirrhosis is characterized by an excessive accumulation of connective tissue, with resultant distortion of the liver vascular bed, shunting of the inflowing portal vein blood through larger vessels and away from the delicate liver sinusoids, directly into the hepatic vein and the general circulation [1,2]. Cirrhosis compromises the synthetic and metabolic function of the liver, bearing the risk of acute-on-chronic-liver failure (ACLF), portal hypertension with extrahepatic collaterals, associated esophageal variceal bleeding, formation of ascites and general immunological dysfunction. The liver connective tissue in the healthy and cirrhotic liver is composed of non-parenchymal cell types, that is, (myo-)fibroblasts and various types of immune cells and of extracellular matrix (ECM). The ECM comprises a multitude of structural and functional molecules, specifically collagens, non-collagenous glycoproteins, glycosaminoglycans and proteoglycans. In a broader sense, the ECM includes cytokines, growth factors and proteases that are attached to it, often in their pro-forms, as well as specific cellular ECM receptors, such as the integrins [2,3]. Moreover, even abundant ECM molecules like collagens and especially certain proteolytic fragments of ECM molecules that are generated during tissue remodeling, angiogenesis and tumor growth, have potent signaling properties that modulate the growth and metastasis of (cancer) cells, angiogenesis, cellular differentiation and immune responses [2,4,5].

Activated myofibroblasts are the main collagen (and general ECM) producing cells in the liver and other organs. They transdifferentiate from hepatic stellate cells (HSCs, liver sinusoidal pericytes) and portal fibroblasts but also to a minor extent from bone-marrow-derived fibrocytes. These activated myofibroblasts are primary target cells for antifibrotic therapies but more recently also for the treatment of (liver) cancer [2].

Clinical management of HCC is complex and depends on the stage of the disease, the remaining liver function, the patient’s comorbidities and available resources and knowhow at the medical center. Liver transplantation is the only potentially curative treatment but available to only few patients with earlier stage HCC. A subgroup of patients can be treated, usually in a palliative intention, by careful liver resection, radiofrequency ablation or chemoembolization. However, the vast majority of patients are diagnosed with advanced stage HCC in a cirrhotic liver, which leaves only limited therapeutic options. Conventional combination chemotherapies are ill tolerated in patients with cirrhosis and show little efficacy [6]. Major advances have been made in the pharmacological treatment of advanced stage HCC. Besides the approval of the novel multikinase inhibitors (MKI) Lenvatinib as an alternative to sorafenib for first line-treatment, second-line treatments have shown promising results [7]. In 2019, the biological ramucirumab (monoclonal antibody against VEGFR2) was approved by the FDA as second-line treatment for HCC in Sorafenib pretreated patients with an alpha fetoprotein (AFP) level >400 ng/mL [8]. In addition, the combination of both biologicals atezolizumab and bevacizumab (monoclonal antibodies against PD-L1 and VEGF-A, respectively) resulted in improved overall survival at 12 month (67.2% vs. 54.6%) and in a better progression-free survival (6.8 vs. 4.3 months) compared to sorafenib [9].

To date, pharmacological approaches in search for new drugs have focused mainly on oncogenic signaling pathways, while the tumor microenvironment (TME), in which tumors develop, has only recently become an important target for anti-cancer therapies. The TME contributes to three key mechanisms of cancers to evade immune recognition—(1) allowing the (epithelial) cancer cells to thrive in a chronically inflamed environment by shifting the micromilieu towards immunosuppression; (2) promoting cancer growth via inducing angiogenesis in a hypoxic environment; (3) facilitating active immune evasion of the cancer cells [10]. The cellular TME has been best studied and first successful therapies have emerged based on the development of checkpoint inhibitors that block immune suppressive signaling between myeloid and T cells [11]. However, this approach has not been very successful for the treatment of HCC [12].

More recently, the connective tissue, that is, the ECM and the nonepithelial cells that are associated with it, has gained increasing interest [13,14,15]. Notably, all solid cancers contain ECM components, even when this is not obvious by routine histology. The role of the ECM is apparent when synthesized by myofibroblasts that are infiltrating the cancer tissue, as in desmoplastic tumors or in the desmoplastic capsule around otherwise stroma-poor cancers. In other epithelial cancers, including most cases of HCC, most of the minor intratumoral ECM can only be visualized with antibodies to certain ECM molecules. In each case the ECM supports the growth of tumor nodules and forms the interface between the cancer and the nontumorous tissues of the body. In advanced cancer, the TME usually mediates immunosuppression and transcriptional alterations in the TME were found to be more predictive for the survival of HCC patients than in the tumor epithelia themselves, underlining a key role of the TME in cancer progression [10]. Importantly, apart from the immune cells in the tumor connective tissue that are skewed towards immunosuppression, the tumor myofibroblasts, that is, the cancer associated fibroblasts (CAF), support cancer growth by secreting pro-cancerous, immunosuppressive and pro-angiogenic ECM components and growth factors. In this vein, analysis of the connective tissue of HCC has revealed several immune- and ECM-related transcriptome signatures derived from CAF that can be correlated with tumor growth and aggressiveness and patient survival [10,16,17,18,19].

## 2. Cancer Associated Fibroblasts

Harold Dvorak coined the phrase that cancer can be understood as “a wound that does not heal” [20]. In analogy with this conception, CAF would generate and maintain “sickly scars” that prevent the cancerous wound from healing. As the most abundant cell type of the tumor microenvironment, CAF support multiple aspects of tumor development and growth by suppression of antitumor immune responses and by remodeling the TME in favor of tumor expansion [21,22,23,24]. Albeit CAF represent a heterogenous cell population of activated (myo-)fibroblasts, they are thought to derive from a variety of different sources, including hepatic stellate cells (HSC, the liver sinusoidal pericytes), (vascular) fibroblasts but to a minor degree also endothelial cells and bone marrow-derived precursors [25,26]. In a mouse model of inflammation-dependent gastric dysplasia, bone marrow-derived mesenchymal stem cells were recruited to the dysplastic stomach and contributed to ~25% of α-SMA + CAF [27]. On the other hand, Arina et al. demonstrated that CAF derive primarily from local fibroblasts and not from cells recruited from outside of the TME [28]. However, solid cell fate-tracing studies are still lacking, casting doubt on the origins of these cells [29]. The progenitors of CAF may vary, depending also on the particular tumor biology and entity and may also differ between mice and humans.

The mesenchymal origin of CAF is reflected by a high expression of α-smooth muscle actin (α-SMA), fibroblast activation protein (FAP), type I collagen and platelet derived growth factor receptor-alpha/beta (PDGFRα/β). Further markers associated with CAF are vimentin and the cell cycle regulating protein FSP-1 (fibroblast-specific protein, S100A4). However, these markers are not exclusively expressed by CAF and markers need to be combined and applied in a context-specific way to distinguish CAF from other (non-)mesenchymal cells [21].

In HCC, as already found in advanced liver fibrosis and in cirrhosis, the ECM of the associated TME is dysbalanced compared to healthy liver tissue, both quantitatively and especially qualitatively and differently in the desmoplastic stroma that surrounds the cancer compared to the usually less abundant intratumoral stroma. Thus, it contains increased levels of different non-fibril forming collagen types (especially types IV, VI, VII, X, XIV, XV, XVI and XVIII) as well as certain glycoproteins and proteoglycans [10,30].

**Figure 1:**
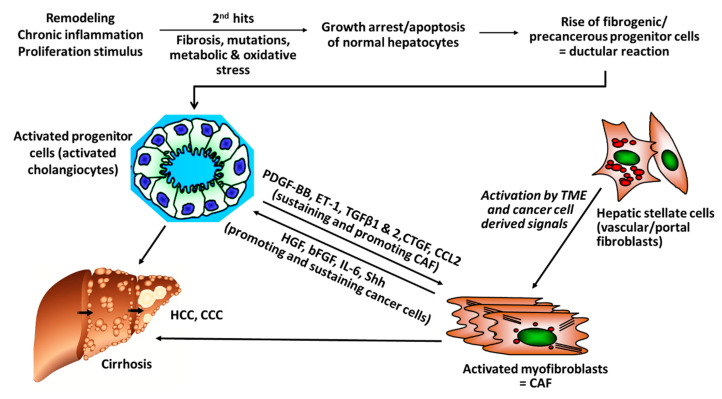
Versatile cross-talks of CAF with activated progenitor cells and HCC. Liver progenitor cells (activated cholangiocytes, oval cells) emerge once the liver is exposed to repetitive and multiple damage, including chronic inflammation of a “wound that does not heal.” The progenitor cells are more resistant to chronic stress and can differentiate into healthy hepatocytes, when the injury subsides but also to fibrogenic and even cancerogenic epithelial progenitors. They engage in a bi-directional crosstalk with stromal cells that include inflammatory cells like TAM (M2-type macrophages) but also activated HSC/myofibroblasts that mutually sustain their growth and expansion, resulting in advanced fibrosis/cirrhosis and/or HCC and CCC. Apart from this pathway for fibrosis that is also prevalent in pancreatic ductal adenocarcinoma (PDAC) and other desmoplastic cancers, additional “second hits” like genetic alterations induced by hepatitis virus integration into the hepatocyte genome, other mutations or metabolic promoters, like in non-alcoholic steatohepatitis (NASH), further promote and sustain hepatic cancer (bFGF, basic fibroblast growth factor; CAF, Cancer-associated fibroblasts; CCL2, CC-Chemokin-Ligand-2 (CCL2); CTGF, connective tissue growth factor; ET-1, Endothelin-1; HGF, hepatocyte growth factor; IL-6 Interleukin 6; PDGF-BB/AB, platelet-derived growth factor BB/AB; Shh; hedgehog signaling pathway).

There is rising evidence from clinical studies that certain genetic signatures of CAF are associated with poor prognosis, for example, after resection of HCC with curative intention, suggesting that stromal components of the TEM, that is, CAF and their synthesis of ECM and growth factors play a pivotal role in cancer progression. For example, a high degree of peritumoral myofibroblast infiltration and a high expression of α-SMA was associated with a significantly higher risk for HCC recurrence post resection [31,32]; osteopontin, a non-collagenous ECM glycoprotein that stimulates the transition of fibroblasts to CAF and also engages in tumor-associated-macrophage (TAM) signaling was found to be highly expressed in solid tumors, including HCC, in correlation to tumor grade, stage, future recurrence and metastasis [33,34,35,36,37]; in this vein, high osteopontin serum levels were associated with poor prognosis, reduced liver function, a worse Child-Pugh score (a clinical-laboratory chemical score determining liver function), shortened disease-free and overall survival [38,39,40,41]. Notably, CAF engage in an extensive bi-directional signaling with hepatic progenitor cells that drive liver fibrosis and can function as cancer stem cells. This mutually supportive interaction illustrates the close link between cirrhosis and liver cancer development. It also underlies similar interactions in other desmoplastic tumors, such as pancreatic, lung adeno- or renal cell carcinoma (Figure 1).

## 3. Stromal Cells Promote Angiogenesis

The angiogenic nature of HCC fosters a vascular network that differs substantially from vessel structures in healthy tissues as tumor vessels display a loss of endothelial fenestration and a high number of vascular pericytes (HSCs) and myofibroblasts (the equivalent of CAF) [42]. HSCs are in immediate proximity to the liver sinusoidal endothelial cells and together with macrophages form a functional unit. They regulate hepatic perfusion, in part by their contractile properties and respond to hepatic injury by transdifferentiation into myofibroblasts, promotion of fibrogenesis and regeneration [32]. HSCs produce numerous angiogenic factors, including VEGF, angiopoietin-1 and angiopoietin-2, which activate the corresponding receptors of endothelial cells to boost their function and promote angiogenesis [43,44,45]. The crosstalk between HSCs and endothelial cells could be demonstrated in in vitro and in vivo studies by co-culture systems and xenograft models employing co-implantation of HSCs and tumor epithelial cells [46,47,48]. VEGF is a strong anti-apoptotic factor for endothelial cells and promotes their proliferation, sprouting and increases vascular permeability resulting in the formation of new vasculature and an improved oxygen and nutrient supply for neighboring (tumor) cells [42]. Ang-1 stabilizes vascular structures after binding to its receptor Tie-2 on endothelial cells [49]. Overexpression of Ang-1/2 is associated with poor differentiation in HCC [50].

Beside the secretion of proangiogenic factors, activated HSCs are also involved in vessel formation. 3D co-cultures of HSCs with endothelial cells revealed a spontaneous organization of the cells, where endothelial cells circled around a core of HSC, forming vessel-like structures [51].

In vitro data revealed that CAF receive proangiogenic signals of tumors to induce neovascularization as conditioned medium of HCC cells stimulated CAF to secrete angiogenic VEGF and bFGF [52,53].

**Figure 2:**
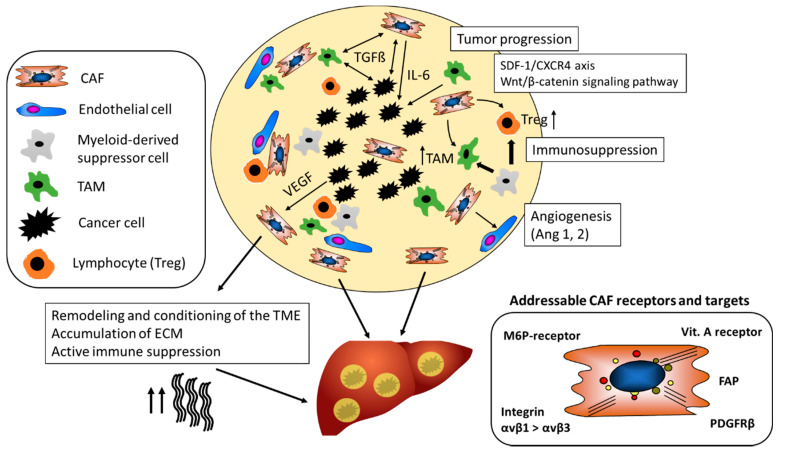
The tumor-promoting role of CAF in the TME. CAF maintain a tolerogenic character of the TME and promote the differentiation of regulatory T-cells (Treg) and tumor-associated-macrophages (TAM). They support tumor neovascularization by stabilizing vessels via secretion of, for example, Ang-1. CAF mediated alterations of the ECM contribute to resistance to chemotherapeutics and checkpoint inhibitors and support tumor growth and metastasis. Different CAF receptors and targets have been identified for cell-specific drug delivery (Ang-1, Angiotensin-1; CAF, Cancer-associated fibroblasts; CXCR4, chemokine receptor type 4; FAP; ECM, extra-cellular matrix; Fibroblast activation protein; PDGFRβ, platelet-derived growth factor beta; M6P-receptor, mannose-6-phosphate receptor; SDF-1, stromal cell-derived factor 1; TGFβ1, transforming growth factor beta 1; VEGF, Vascular endothelial growth factor).

As illustrated in Figure 2, CAF (and macrophages) are instructed by the tumor cells to secrete proangiogenic factors.

## 4. The Secretome of CAF Supports the Growth of HCC

TGFβ1 is a pleiotropic cytokine with an important role in fibrogenesis, a cofactor of cancer development and a key molecular player in the CAF-governed tumor stroma [54]. TGFβ1 also contributes directly to hepatocarcinogenesis via auto- and paracrine activities in the cancer epithelial cells. Thus, TGFβ1-dependent signals induce epithelial-mesenchymal transition that promotes survival and aggressiveness of liver cancer cells and that increases their resistance to chemotherapy, hypoxia, heat treatment and starving conditions [55,56,57,58]. As an example, CAF derived TGFβ1 activates the smad-2/3 and the beta-catenin signaling pathway within premalignant hepatocytes, which in turn drives multiple cellular processes that are involved in the initiation, growth, survival, migration, differentiation and apoptosis of HCC (Figure 2) [59].

Beside TGFβ1, CAF secrete strong mitogenic factors for HCC cells, such as hepatocyte growth factor (HGF) and epiregulin that promote tumor growth. Inhibition of HGF in vitro decreases proliferation and invasive potential of several hepatoma cell lines [60]. Under physiological conditions HGF is mainly produced by activated HSCs and to a minor degree by endothelial and myeloid cells. Notably, HSCs show a massive upregulation of HGF expression in human liver fibrosis and liver cancer and HGF is stored in the fibrotic and desmoplastic cancer ECM, binding specifically to certain collagens [61,62]. HGF promotes HCC via activation of the c-Met receptor and both the FRA1/HEY1 and MAPK/ERK dependent pathways [63]. Beside HCC, HGF/c-MET signaling plays also a role for other solid cancers such as breast, lung, stomach and prostate cancer. Cabozantinib, a TKI with activity against c-MET, VEGFR2 and RET reduced tumor growth in vitro and in several mouse xenografts models in vivo [63]. A draw-back of these xenograft studies is the lack of T cells which prohibits to draw conclusions of efficacy in real in vivo tumor scenarios. Further, several antibodies (e.g., rilotumumab, ficlatuzumab) addressing the HGF-cMET axis are tested in advanced clinical trials for several solid cancers [64].

CAF can engage multiple mechanisms that support cancer growth and subvert the host’s defense tumor cells. The following is an incomplete selection that illustrates only few such mechanisms. CAF-derived IL-6 enhances progranulin expression by HCC cells which promotes the malignancy of HCC cells by activating mTOR signaling, a nutrient sensing pathway that supports malignant growth. Further, IL-6 triggers the activation of the IL-6/JAK/STAT3 pathway which generally enhances proliferation, survival, invasiveness and metastasis of tumor cells, while strongly suppressing the antitumor immune response (Figure 2) [65].

The profibrotic tissue inhibitor of metalloproteinases 1 (TIMP-1) is an endogenous inhibitor of various matrix metalloproteinases (MMPs), which are involved in the degradation and turnover of the ECM in fibrosis but also in cancer growth and metastasis [66]. High levels of TIMP-1 in HCC are correlated with poor prognosis and advanced TNM stage. In vitro, TIMP-1 promotes cell proliferation and migration of patient derived CAF by activation of ERK1/2 kinases [67]. Further, CAF inhibit apoptosis of Huh7 hepatoma cells via SDF-1/CXCR4/PI3K/AKT/mTOR signaling and further promote proliferation of the malignant hepatocytes [68]. Similarly, CAF support the growth of precancerous progenitor cells, which develop from cholangiocytes under repetitive damage and chronic inflammation [69]. In turn, signaling of CAF with progenitor cells is bilateral, thus, activated cholangiocytes maintain also the growth and proliferation of CAF (Figure 1) [70,71,72,73].

## 5. CAF Decrease Immune Surveillance

Several lines of evidence suggest that CAF can have a strong suppressive effect on immune cells and, thus, promote tumor cell escape and proliferation in HCC, CCC and other solid tumors, such as pancreatic duct adenocarcinoma (PDAC). Co-transplants of HSC with tumor cells in immunocompetent mice inhibited lymphocyte infiltration (CD3-, CD4- and CD8-positive cells) in the tumor, while the immune response was significantly higher when tumors were transplanted without HSC [74]. Immunosuppression by HSC was accompanied by an increased apoptosis of infiltrating mononuclear cells and enhanced expression of B7H1 (PD-L1) and FoxP3-positive immunosuppressive Treg cells (CD4+, CD25+) in the TME (Figure 2) [74]. Beside immunosuppression by T-cells, myeloid-derived suppressor cells (MDSCs), similar to TAM contribute to the immunosuppressive effect in HCC and suppress not only the adaptive but also the innate immune response (Figure 2) [75]. In an orthotopic liver tumor mouse model, HSC induced tumor resident MDSC from their bone-marrow precursors via COX2-PGE2-EP4 signaling and stimulated their immunosuppressive activity via secretion of iNOS, Arg-1 and IL-4Rα [76].

Beside cytokine signaling, cell-cell contact was found to be crucial for the immunosuppressive effect of CAF. Co-cultures of human monocytes with LX-2 HSC changed their gene expression profile from an inflammatory to an immunosuppressive signature, namely, upregulation of immunosuppressive (CD15 and CCR2) and downregulation of inflammatory (CD86) markers [77]. This induction of the transdifferentiation of CD14+ monocytes to MDSC did only occur in a contact-dependent manner and was abrogated by inhibiting cell-cell interaction through blockage of the hyaluronic acid (an ECM component) receptor CD44 [78].

Apart from MDSC and as mentioned before, TAM represent the most abundant immune cells in the TME and engage in a bilateral cross talk with immune cells, cancer epithelia and CAF (Figure 2). High numbers of TAM correlate with poor prognosis not only in HCC but also other cancers [79,80]. TAM secrete an excess of some pro-inflammatory (e.g., IL-6, TNF-α), several anti-inflammatory (e.g., IL-10, TGFβ1) and pro-angiogenic (e.g., VEGF, FGF-2) factors and TME remodeling matrix metalloproteinases (MMPs), which together promote cancer growth, survival and metastasis. Similar to CAF, TAM derived IL-6 amplifies undirected, that is, not tumor-targeted, inflammatory responses and promotes HCC via enhanced STAT3 signaling [81].

Besides direct effects on hepatocarcinogenesis, as described earlier, TGFβ1 is a strong suppressor of antitumor immunity by inhibition of natural killer cells, dendritic cells, macrophages, neutrophils, CD8+ and CD4+ effector cells, and, hence, appears to be an important common denominator of CAF, fibrosis and TAM in terms of immunosuppression [82].

CAF build a physical barrier around the tumor as they are important generators of ECM in the TME and, thus, also contribute to tissue stiffness. For example, the collagen rich tumor capsule blocks the influx of novel immune cells into the TME and therefore dampens the immune response towards tumors (Figure 2) [83]. Further, the stiffness of the surrounding tumor tissue has an impact on angiogenesis. Thus, activated metastasis-associated fibroblasts increased tissue stiffness, which in turn triggered angiogenesis and promoted anti-angiogenic therapy resistance. Antihypertensive drugs targeting the renin-angiotensin system inhibited activation and contraction of HSC/CAF, thereby reducing stiffening and improving the antiangiogenic effect of bevacizumab in a model of liver metastases [84].

## 6. CAF as Target in Anti-Stromal Cancer Therapy

The synergistic cross-talk between cancer cells, CAF and immune cells in the TME that together contribute to the progression of cancer have led to the design of preclinical and recently also clinical studies for anti-stromal therapy that would overcome the tumor-resistant environment in epithelial cancers. CAF-directed therapy aims at either at their ablation by interfering with their survival or at “normalizing” them by interfering with secreted pro-tumorigenic signals [85]. The following discusses a selection of promising drug targets and candidates for CAF-based therapies in solid cancers, with special relevance for HCC.

***Fibroblast activation protein (FAP)*** is considered to be a specific marker for CAF. It is a membrane-bound serine dipeptidyl peptidase of 760 amino acids with a large extracellular (amino acids 26–760) and a small intracellular domain [86]. FAP is almost absent in healthy tissues but highly expressed by up to 90% of myofibroblasts of carcinomas of the breast, colorectum, pancreas, lung, bladder and ovaries [86]. In cirrhotic livers and liver cancer FAP activity is upregulated 14 to 18 fold vs. healthy livers, respectively [87,88]. Under homeostasis, FAP does not appear to play an essential physiological role, since FAP knockout mice are viable and display no overt defects [89]. In knockout mice for DPP4, the enzyme most similar to FAP, liver fibrosis was attenuated [90].

FAP supports tumor growth in multiple ways. One of the most consistent finding is that FAP promotes tumor cell proliferation, migration and invasion, all of which favor tumor growth. The underlying mechanisms are still debated. One partly supported hypothesis is that FAP, with its exo- and endo-dipeptidyl peptidase activity that is directed to certain ECM proteins, remodels the ECM for increased capability to support (cancer) cell growth [86]. Several lines of evidence indicate that FAP expressing myofibroblasts directly induce immunosuppression, while the exact mechanism is still unclear. In a transgenic mouse model for pancreatic ductal adenocarcinoma (PDAC), where FAP expressing cells were depleted by injecting diphtheria toxin, CAF depletion led to reduced tumor growth, which was mediated by enhanced CD4+/CD8+ T cell activity. Further, FAP ablation improved the therapeutic benefit of check-point inhibitor treatment with anti-PD-1 and to a lesser extent with anti-CTLA4, suggesting that FAP contributes to the resistance of PDAC to this treatment [86,91]. FAP positive CAF secrete high levels of CCL2 which in turn addresses the CCL2 receptor (CCR2) on circulating MDSC, activating immunosuppressive STAT3 signaling and leading to an enrichment of MDSC in tumor tissues. In CCL2 knockout mice, co-inoculation of FAP+ CAF with PDAC tumor cells resulted in comparable tumor growth compared to tumor inoculation with FAP- CAF, while no differences in numbers of MDSC were observed [92]. Lentiviral shRNA mediated knockdown of FAP reduced cell proliferation, together with induction of cell cycle arrest of cancer cells in a xenograft mouse model of oral squamous cell carcinoma (OSCC), underlining the relevance of FAP-CCL2-signaling as therapeutic target in solid tumors [93].

There is also evidence that FAP supports cancer angiogenesis. In a xenograft mouse model for breast cancer, FAP+ breast cancer cell lines showed faster growth of highly vascularized tumors, albeit this proliferative advantage was not observed in vitro [94]. In biopsies of gastric cancer, high FAP expression correlated with micro-vessel density vs gastric cancers with lower FAP expression [95]. The underlying mechanism how FAP acts proangiogenic is still debated. One hypothesis suggests that FAP processes neuropeptide Y, which can be pro-angiogenic, promoting endothelial cell migration and tube formation on a basement membrane matric (Matrigel). On the other hand, the pro-angiogenic effect might be related to MMP-9, a known proangiogenic MMP, rather than FAP itself, as MMP-9 is often co-expressed with FAP [96].

Despite promising results in preclinical models, clinical studies evaluating the FAP inhibitory antibody sibrotuzumab (BIBH 1, Boehringer Ingelheim, Germany, a humanized version of a murine anti-human FAP mAb) were disappointing in colon cancer [97]. On the other hand, the depletion of FAP+ stromal cells (CAF) demonstrated a therapeutic benefit synergistic with immunological checkpoint inhibitors in PDAC [98]. Ongoing clinical trials are evaluating the therapeutic value of chimeric antigen receptor (CAR) T cells engineered to target FAP expressing CAF. Further, one bispecific antibody and fusion protein, RO6874813 (Anti-FAP/anti-death receptor 5, DR5) and RO6874281 (anti-FAP/ interleukin-2) are currently tested in clinical phase 1 and 2 studies, respectively [98].

***Targeting the CAF secretome,*** apart from direct targeting of CAF, represents another promising approach. CXCL12 (SCDF, fractalkine) was identified as an immunosuppressive chemokine, which is secreted by CAF. Inhibition of CAF derived CXCL12 in a transgenic mouse model for PDAC synergized with α-CTLA-4 and α-PD-L1 inhibitors to induce an anticancer immune response [91].

***TGFβ1*** (and less well define other TGF-β family members) is a key cytokine that is highly secreted by activated HSC and CAF promoting fibrosis, epithelial mesenchymal transition of cancer cells (EMT) and TEM immune suppression, as mentioned before. Several clinical trials applying activity blocking TGFβ antibodies, TGFβ kinase inhibitors or TGFβ antisense oligonucleotides have been conducted. Galunisertib (a small molecule TGFβ1 type I receptor, Alk5, inhibitor) has yielded promising results when combined with Sorafenib (a multi-tyrosin kinase inhibitor) and is currently tested together with Nivolumab (a human IgG4 monoclonal antibody blocking PD-1) in a clinical phase 2 study with HCC patients [99,100].

There are red flags for targeting CAF in general, as their complete depletion can enhance malignancy of tumors. The depletion of a-SMA+ myofibroblasts (a subgroup of CAF) led to more invasive and undifferentiated tumors in a transgenic mouse model for PDAC, suggesting that the function of a-SMA+ CAF depends greatly on the tumor microenvironment, cancer type or the preclinical model [101]. However, the expression of a-SMA and FAP appears to characterize two distinct phenotypes of activated HSC/CAF, possibly calling for selective ablation or inhibition of FAP-positive CAF [102,103,104].

***CCL2***, a major chemoattractant and activator for TAM and MDSC, is a prominent product of activated HSC in fibrosis and of CAF [92]. It is therefore an attractive target for nanoparticular therapies that are either based on delivery of small molecule inhibitors, siRNA or ASO.

***The limitations of current therapies targeting CAF*** are that neither cellular markers nor the secreted cytokine/chemokine pattern can be exclusively attributed to CAF, bearing the risk of CAF based therapies to induce unwanted, even tumor promoting side effects, as other cancer-inhibiting cell types may be affected. An example, are certain TGFβ targeting drugs, such as AP-12009, anti-TGFβ2, lerdelimumab, 2G7, that advanced into clinical phase 3, since TGFβ is a double edged sword in the context of solid cancers. Thus, TGβ can display both pro- and, via its anti-proliferative activity, also anti-tumoral effects [105]. Further, all the above described therapies are based on systemic drugs that need to be applied at relatively high dose to reach pharmacologically relevant concentration in CAF and the TME. Since the liver can be easily targeted with a large variety of nanoparticles (even without organ specific targeting), nanoparticle-based drugs and formulations have a high potential to overcome the limitations of systemic drugs in liver cancer.

## 7. Cell Specific Targeting of CAF with Nanoparticles

Most groups aimed to target activated HSC in the context of liver fibrosis (see Table 1 and Table 2), while there is limited data of cell-specific targeting of CAF with nanoparticular systems. However, due to partly identical immune signaling in fibrosis and cancer and the similarity of both activated HSC and CAF that are part of a spectrum of myofibroblast, the gained body of knowledge and identified targets in fibrosis also represent a promising platform for CAF specific targeting in HCC. Here we discuss the current key molecular targets and some pivotal studies of drug conjugates (Table 1) and nanoparticles (Table 2) for HSC/CAF-specific drug delivery.

### 7.1. M6P/Insulin-Like Growth Factor II (M6P/IGFII) Receptor

The M6P/insulin-like growth factor II receptor (M6P/IGFIIR) is a transmembrane glycoprotein with a large extracellular domain. The receptor is involved in the removal and degradation of extracellular proteins after their endocytosis and in signal transduction by G-protein coupled receptors. Several IGFIIR ligands with and without mannose-6-phosphate (M6P) were identified, including granzyme B, renin, latent TGFβ1, thyroglobulin, proliferin, retinoic acid or urokinase-type plasminogen activator receptor [125]. The IGFIIR is upregulated in activated HSC and is strongly expressed on HSC/CAF during hepatic carcinogenesis [51].

Beljaars et al. were the first to employ the M6P/IGFIIR for HSC specific targeting using human serum albumin (HSA) as carrier for both the covalently-linked M6P-ligand [126]. The HSA-M6P carriers showed a rapid accumulation in liver and a high cellular uptake in HSC of fibrotic livers, reaching almost 50% of the intravenously applied dose. The variant with the highest ratio of M6P to HSA (28:1) achieved the highest uptake in activated HSC, demonstrating that the amount of HSA-bound M6P is crucial for effective cell-specific targeting. In follow-up studies, M6P-HSA was conjugated to several drugs with anti-proliferative or antifibrotic potential, including doxorubicin, mycophenolic acid, 15-deoxy-Δ12,14-prostaglandin J2 (15dPGJ2) and so forth. (see also Table 1) [127,128,129]. All drug conjugates demonstrated superior antifibrotic activity in vivo compared to systemic application of their unconjugated counterparts. The same group also derivatized liposomes, consisting of 1-palmitoyl-2-oleoyl-sn-glycero-3-phosphocholine (POPC), 1,2-dioleoyl-sn-glycero-3-phosphoethanolamine-N-4-(maleimidophenyl)butyramide (MPB-PE) and Cholesterol (Chol), with surface M6P [130]. The M6P-HSA-liposomes accumulated rapidly in the fibrotic liver and were efficiently taken up by HSC, Kupffer cells (liver resident macrophages) and endothelial cells. Polyinosinic acid, a competitive inhibitor of scavenger receptors other than IGFIIR, abrogated the uptake in Kupffer cells and liver sinusoidal endothelial cells, while the uptake in HSC remained, indicating that HSC specific uptake can be further enhanced by co-application of this nontoxic polysaccharide (Table 2) [124].

### 7.2. PDGFRβ

The noncovalently linked dimeric PDGF-BB (and PDGF-AB) is a key cytokine to drive the proliferation of activated HSC in liver fibrosis and the desmoplastic TME. Its cellular receptor, the dimeric PDGFRβ, is strongly upregulated on activated HSC. Poelstra et al. devised a cyclic peptide (pPB: C∗SRNLIDC∗) that contains the 3 central amino acids of PDGF-B and that binds to the PDGFRβ with high affinity [131].

Similar to HSA-M6P, pPB was conjugated to HSA, serving as guiding ligand (HSA-pPB) for cell specific drug delivery to HSC. Activation of the Rho-kinase (ROCK) in HSC has been identified as key mechanism for the establishment of portal hypertension (PTH) and fibrogenesis in liver fibrosis [107]. Thus, the ROCK-inhibitor Y-27632 (Y27) was conjugated to HSA-pPB (Y27-HAS-pPB) to achieve higher drug load in HSC (Table 1). At a dose of 1 mg/kg Y27-HSA-pPB hemodynamic parameters (portal pressure and, hepatic vascular resistance) significantly improved (33% PP and 57%, respectively vs. controls) in bile duct ligated fibrotic rats compared to untreated controls. However, levels of collagen and the fibrosis surrogate marker a-SMA did not differ between treated and untreated rats after single application of the drug conjugate [107].

Th pPB peptide was also incorporated into the outer surface of liposomes loaded with interferon gamma (IFNγ), a cytokine that can induce fibrolytic responses in fibroblasts and macrophages [115]. These pPB- IFNγ-liposomes demonstrated a significantly improved antifibrotic effect compared to systemic IFNγ in a murine model of thioacetamide-(TAA)-induced parenchymal liver fibrosis [115]. Moreover, as shown before with pPB- and IFNγ loaded intravenously injected HSA [112], the IFNγ-loaded pPB-liposomes were as efficient as high dose systemic IFNγ but lacked the unwanted side effects of the systemic treatment like fever and leukopenia [115]. More recently, liposomes were decorated with pPB and loaded with anti-HSP47 (heat shock protein 47) siRNA. HSP47 is a molecular chaperone in the endoplasmic reticulum with a high affinity for procollagens and therefore an interesting target for the treatment of liver fibrosis. SiRNA loaded pPB-liposomes demonstrated a significant inhibitory effect on TAA-induced hepatic fibrotic mice, while the antifibrotic activity was comparable to their unconjugated counterparts [116].

### 7.3. Vitamin A/Retinol Binding Protein Receptor

HSC are the major vitamin A storing cells in body. They take up vitamin A from the circulation and especially from neighboring hepatocytes through receptors for retinol binding proteins (RBP) and store it as retinol esters. Therefore, vitamin A, as targeting ligand, coupled to liposomes (VitA-liposomes) should enhance uptake in HSC (Table 2) [117]. In vitro cellular uptake of fluorescence-labeled siRNA/VitA-liposomes in primary HSC revealed that the highest mean fluorescence intensity in HSC was observed at a molar vitamin A/liposome ratio of 2:1. Five treatments with anti-HSP47 siRNA loaded VitA-liposomes (corresponding to 0.75 mg/kg siRNA) almost completely prevented liver fibrosis in 3 models and prolonged survival in rats with otherwise lethal dimethylnitrosamine-induced liver fibrosis [117].

Qiao et al. reported the synthesis and in vivo evaluation of VitA-lipoplexes consisting of amphiphilic cationic hyperbranched lipidoids and helper lipoids (cholesterol-polyethylene glycol-vitamin A, Chol-PEG-vitamin A) as siRNA carrier for HSC specific targeting (Table 2) [118]. In co-culture experiments for cellular uptake, fluorescence-labeled and siRNA loaded VitA-lipoplexes were efficiently taken up by the rat HSC-T6 cell line, while uptake was low in macrophages (RAW cell line). SiRNA loaded VitA-lipoplexes demonstrated a significant knockdown for their target transcripts col1a1 or timp1 (encoding Procollagen α1(I) and TIMP-1, respectively) in TGFβ1 stimulated LX-2 cells (human HSC cell line). After intravenous injection, near infrared dye-labeled VitA-lipoplexes showed an enhanced accumulation in fibrotic compared to non-fibrotic livers or control lipoplexes without targeting ligand. Morphometrical collagen quantification revealed that VitA-derivatized lipoplexes carrying both anticol1a1 and antitimp1 siRNA reduced hepatic collagen accumulation more efficiently (~2-fold) than lipoplexes carrying either anticol1a1 or antitimp1 siRNA alone [118]. A caveat to the efficacy of vitamin A loaded nanoparticles for HSC targeting in advanced fibrosis is the finding that with fibrogenic activation, HSC lose their vitamin A stores and likely their ability to ingest vitamin A via RBP receptors. Moreover, studies on HSC/CAF targeting in HCC are lacking.

### 7.4. Integrin αvβ3

One of the hallmarks of activated HSC is their increased expression of integrin αvβ3. Therefore, liposomes endowed with a cyclic peptide that displays a high affinity for the integrin αvβ3 (cRGDyK) were developed (Table 2) [132]. Fluorescence labeled cRGDyK-liposomes showed a high specific uptake in the HSC-T6 cell line, while their in vitro uptake in primary hepatocytes, Kupffer cells and liver sinusoidal endothelial cells was low. To date this construct has only been used for in vivo imaging showing a significantly higher accumulation of the carrier in fibrotic livers compared to the unguided control carriers, while the biodistribution of cRGDyK-liposomes was equal to unguided control carriers in healthy mice with non-fibrotic livers [119]. Here, drug delivery studies are lacking. Moreover, in vivo and functionally the αvβ3 integrin is broadly upregulated in different nonparenchymal cell types in fibrosis (HSC, macrophages, endothelial cells) reducing its putative HSC/CAF specificity [73]. Targeting the highly HSC-specific integrin αvβ1, for which specific inhibitors have become available only recently, is an attractive alternative [133].

### 7.5. CXCR4

CXCR4 is a chemokine receptor overexpressed in activated HSC. One of its ligands, CXCL12 (SDF-1, fractalkine) has been shown to promote fibrogenic activation of HSC [120]. CXCR4 guided polyplexes composed of poly(lactic-co-glycolic acid) (PLGA), 1,2-dioleoyl-sn-glycero-3-phosphocholine (DOPC) and 1,2-distearoyl-sn-glycero-3-phosphoethanolamine-N-maleimide(polyethylene glycol)-2000 (DSPE-PEG-maleimide), equipped with a CXCR4 antagonist peptide for cell-specific targeting of HSC in liver fibrosis, have been developed (Table 2) [120]. Co-formulations of the multi-TKI Sorafenib together with AZD6244, a mitogen activated kinase (MEK1/2) inhibitor, lead to a strong suppression of HSC activation in vitro, while formulations with Sorafenib alone even induced HSC activation and survival via extracellular signal-regulated kinases (ERK) activation. Biodistribution of near-infrared labeled CTCE9908 guided NPs revealed a significantly (*p* < 0.001) increased uptake in fibrotic livers compared to comparative doses of free drug or non-targeted control-NPs in the fibrotic livers of CCl4-treated mice. Co-formulations of Sorafenib and AZD6244 decreased liver fibrosis and suppressed angiogenesis in CCl4 fibrotic mice (each 2-3-fold), while free drugs and drugs loaded in unguided co-formulation were ineffective. Further, systemically administered co-formulations of Sorafenib and AZD6244 prevented HCC in DEN- and CCl4-induced liver fibrotic mice, with tumor incidence in the treated group being reduced 7-fold compared to the placebo-treated control group.

### 7.6. Unguided Nanoparticles with HSC/CAF Specific Cargos

The liver represents an organ which shows a high passive accumulation for nanoparticles due to—(1) its high perfusion (25% of the cardiac output, while it constitutes only 2.5% of body weight) coupled with a high capacity to remove nutrients, metabolites and particulate material from the bloodstream (mainly via liver resident macrophages, that is, Kupffer cells and sinusoidal endothelia); (2) In line with the enhanced permeability and retention (EPR) effect in tumors, fenestrations in the sinusoidal capillaries allow for foreign particles up to a diameter of ~100 nm to be trapped in the space of Disse (between the sinusoidal endothelia and the hepatocytes, where HSC and Kupffer cells are located). Thus, even without specific targeting ligands, nanoparticles of appropriate size often come in close proximity to HSC, Kupffer cells and sinusoidal endothelia. This applies to lipoplexes of appropriate size and zeta potential and to nanohydrogel particles (NHPs) (Table 2) [116,121,122,123]. NHPs are well-defined nanocarriers that are suitable for delivery of small anionic molecules including siRNA. They consist of biocompatible amphiphilic co-block polymers with a covalently stabilized inner core [134]. In the second generation of NHPs in vivo tolerance was improved by the introduction of an acid-labile core, yielding biodegradable (bio-)NHPs [121]. After loading with siRNA, surface charge (zeta-potential) of the carriers was neutral, avoiding unspecific interactions with serum proteins. Anticol1a1 siRNA loaded NHPs as well as bio-NHPs (2 mg/kg siRNA) demonstrated a robust antifibrotic effect in CCl4 liver fibrotic mice, with a 50% reduction of liver collagen [121,122]. After intravenous injection, near infrared-labeled carriers rapidly accumulated in the fibrotic livers, with a comparable cellular uptake in activated (a-SMA+) HSC and a lack of toxicity after repeating intravenous administration. Therefore, both lipid and defined non-lipid nanocarriers can be designed to efficiently target siRNA to the liver and here predominantly nonparenchymal cells and HSC/CAF, even without cell-specific targeting ligands. Simultaneous uptake by different nonparenchymal cells, especially HSC/CAF, macrophages and endothelial cells may either not play a role, as long as the target gene is only expressed by HSC/CAG (e.g., col1a1) or be synergistic if the targeted gene or protein generally promotes immunosuppression in cells of the TME (e.g., stat3, IL-10, TGFβ1). If further specificity is needed, the NHPs can be decorated with cell specific ligands, such as shown for mannose surface decoration for cell-specific targeting of TAM (that express the mannose receptor CD206) in liver fibrosis, which has been shown by us in the current issue of Cells [135].

## 8. Conclusions and Outlook

CAFs and their ECM products have previously been considered as silent bystanders in cancer biology and deemed of minor relevance for cancer treatment and drug discovery. Only recently, their relevance, not only in stroma rich tumors, such as CCC, subtypes of HCC and PDAC but also in stroma poor cancers has emerged. CAF were identified as one of the major tumor-promoting cellular players in the desmoplastic tumor stroma. They support cancer development, growth, metastasis and resistance to chemo- or checkpoint inhibitor therapy by a multitude of mechanisms, including ECM remodeling, angiogenesis, active immunosuppression and secretion of tumor promoting cytokines, chemokines and growth factors such as EGF, HGF, CCL2, TGFβ1 and VEGF. CAF can be addressed via specific surface receptors, especially for targeted nanoparticular drug delivery. FAP has been identified as a cell-specific marker for CAF, which also functionally contributes to the protumor activity of CAF. Several nanoparticles and nanoligands were tested successfully to deliver drugs to activated HSC/myofibroblasts, the equivalent of CAF in liver cancer. Here, surface modified nanocarriers with a cyclic peptide to the PDGFRβ or M6P that target IGFRII achieved a robust nanoparticle-based drug delivery to activated HSC in vivo. Moreover, unguided nanohydrogel particles and lipoplexes loaded with siRNA demonstrated a high in vivo uptake and functional siRNA delivery in activated HSC, highlighting that hepatic CAF can also be targeted by well-devised carriers with optimized physicochemical properties. Taken together and with the comparable role of HSC, CAF and the stroma in the development of liver fibrosis/cirrhosis and liver cancer, CAF/HSC have become an attractive target for the development of stroma-based therapies.

## Figures and Tables

**Table 1:** Nanoligands for cell specific drug delivery to HSC.

Addressed Target	Cell-Specific Ligand	In Vivo Cellular Uptake	Coupled/ Encapsulated Drug	In Vivo Therapeutic Effect	Nano Carrier	Size (Zeta Potential)	Reference
Collagen type VI receptors	Cyclic peptide	Activated HSC	Not reported	Not reported	HSA	Not reported	[106]
PDGFRβ	Cyclic peptide	HSC	ROCK-inhibitor Y-27632	Lowers portal pressure	HSA	Not reported	[107]
PDGFRβ	Cyclic peptide	Not reported	None but suitable for delivery of protein-based drugs	Not reported	HSA—polymeric microspheres	~22 μm	[108]
PDGFRβ	Cyclic peptide	Myofibroblasts	Erlotinib (epidermal growth factor receptor inhibitor)	Improved antitumor activity, reduced in vivo toxicity (hepatotoxicity)	Silica nanoparticles	~200 nm	[109]
PDGFRβ	Cyclic peptide	HSC, myofibroblasts	IFNγ peptidomimetic (without extracellular recognition domain)	Improved antifibrotic effect (compared to free IFNγ in CCl4 fibrotic mice)	IFNγ coupled to cyclic peptide	Not reported	[110]
PDGFRβ	Cyclic peptide	Tumor pericytes	IFNγ	Improved anti-tumor effect via inhibition of angiogenesis (compared to unguided IFNγ)	HSA	Not reported	[111]
PDGFRβ	Cyclic peptide	HSC	IFNγ	Improved antifibrotic effect (compared to free IFNγ in CCl4 fibrotic mice)	IFNγ coupled to cyclic peptide	Not reported	[112]
M6P receptor	M6P	HSCs, Myofibroblasts	losartan	Antifibrotic effect vs. free losartan	HSA	Not reported	[113]
M6P receptor	M6P	HSC, >LSEC; no colocalization with macrophages	TGFβ receptor 1 (ALK5) inhibitor LY-36494	Improved antifibrotic activity (reduction of collagen III and fibronectin)	HSA	Not reported	[114]

CCl4, carbon tetrachloride; HSA, human serum albumin; HSC, hepatic stellate cell; LSECs, liver sinusoidal endothelial cells; PDGFRβ, Platelet-derived growth factor receptor beta; M6P, mannose-6-phosphate; ROCK, Rho kinase inhibitor; TGFβ, Transforming growth factor beta.

**Table 2:** Nanocarriers for cell-specific drug delivery to HSC.

Addressed Target	Cell-Specific Ligand	In Vivo Cellular Uptake	Encapsulated Drug	In Vivo Therapeutic Effect	Nano Carrier	Size (Zeta Potential)	Reference
PDGFRβ	Cyclic peptide	HSC	IFNγ	Improved antifibrotic effect vs untargeted IFNγ	Liposomes	~83.5 nm	[115]
PDGFRβ	Cyclic peptide	HSC	antiHSP 47 siRNA	Improved antifibrotic effect vs control siRNA/carriers in TAA fibrotic mice	Lipoplexes	~110 nm (~0 mV)	[116]
Receptors for retinol binding protein	Vitamin A	HSC, (macrophages)	antiHSP47 siRNA	Improved antifibrotic effect vs control siRNA/carriers in liver fibrotic rats	Liposomes	~150 nm	[117]
Receptors for retinol binding protein	Vitamin A	HSC	anticol1a1 and antiTIMP-1 siRNA	Antifibrotic effect vs scrambled siRNA loaded carriers in CCl4 fibrotic mice	Lipoplexes	~140 nm (~−12.9 mV)	[118]
Integrin αvβ3	Cyclic RGD peptide	Activated HSC (+++), Kupfer cells and LSECs (++), biliary cells (+), hepatocytes (+)	Vismodegib (hedgehog inhibitor)	Improved antifibrotic effect vs control drug/empty carrier in BDL fibrotic mice	Liposomes	~80 nm, (~−24.8 mV)	[119]
CXCR4	CXCR4 antagonistic peptide (CTCE9908)	HSC	Sorafenib/multi-tyrosine-kinase inhibitor	Antifibrotic and antitumor effect in mice with CCl4-induced fibrosis, HCC and PDAC	Liposomes	~140 nm	[120]
No specific target	Unguided carriers	HSC (+++), Kupffer cells and LSEC (++), hepatocytes (+)	Anti-col1a1 siRNA	Antifibrotic effect vs scrambled siRNA loaded carriers in CCl4 fibrotic mice	Cationic nanohydrogel particles	~40 nm (~0 mV)	[121,122]
No specific target	Unguided carriers	HSC (+++), Kupffer cells/macrophages (++), hepatocytes (+), LSEC (+)	Anti-col1a1 siRNA	Antifibrotic effect vs scramble siRNA loaded nano- carriers in CCl4 and MDR2-/- fibrotic mice	Lipid cationic nanoparticles		[123]
M6P receptor	M6P	HSC > Kupffer cells > LSEC	No drug reported	No effect reported	Liposomes	~102 nm	[124]

CCl4, carbon tetrachloride; col1a1, collagen 1a1 gene; CXCR4, chemokine receptor type 4; ERK, extracellular signal-regulated kinases; HSC, hepatic stellate cells; HSP 47, heat shock protein 47; IFNγ, Interferon gamma; LSECs, liver sinusoidal endothelial cells; MEK, mitogen-activated protein kinase; M6P, mannose-6-phosphate; PDGFRβ, Platelet-derived growth factor receptor beta; TAA, thioacetamide; TIMP-1, TIMP tissue inhibitor of metallopeptidases 1.

## Abbreviations

ACLF	acute-on-chronic-liver failure
Arg-1	arginase-1
AKT	protein kinase B
ASO	antisense oligonucleotides
a-SMA	alpha-smooth muscle actin
bFGF	basic fibroblast growth factor
CAF	Cancer-associated fibroblasts
CCL2	CC-Chemokin-Ligand-2 (CCL2)
CCl4	Carbon tetrachloride
CXCR4	chemokine receptor type 4
CTGF	connective tissue growth factor
COX2	Cyclooxygenase-2 (COX-2)
c-MET	tyrosine-protein kinase Met
COX2-PGE2-EP4	Cyclooxygenase-2-Prostaglandin E2-Prostaglandin E2 receptor 4
DEN	di-ethyl-nitrosamine
ET-1	Endothelin-1
ERK	Extracellular signal-regulated kinases
EPR	enhanced permeability retention
Erk1/2	extracellular signal-regulated kinases
FAP	Fibroblast activation protein
FLT3	FMS-like tyrosine kinase 3
FRA1	Fos-related antigen 1
HCC	Hepatocellular carcinoma
HGF	hepatocyte growth factor
HEY1	hairy/enhancer-of-split related with YRPW motif protein 1
IGFRII	insulin-like growth factor type II receptor
IL-6	Interleukin 6
IL-4R	Interleukin 4 receptor
iNOS	Nitric oxide synthases
IFNγ	Interferon gamma
JAK	janus kinase
M6P	mannose-6-phosphate
MAPK	mitogen-activated protein kinase
MDR2	multidrug resistance protein 2
MDSC	Myeloid-derived suppressor cells
MMP-9	Matrix metallopeptidase 9
TAM	Tumor-associated macrophages (TAMs)
mTOR	mechanistic Target of rapamycin
PDAC	pancreatic ductal adenocarcinoma
PDGFR(β)	platelet-derived growth factor receptor β
PDGF-BB/AB	platelet-derived growth factor BB/AB
PI3K	phosphoinositide 3-kinase
PGE2-EP4	Prostaglandin E2-Prostaglandin E2 receptor 4
PD-L1	Programmed cell death 1 ligand 1
SDF-1	stromal cell-derived factor 1
Shh	hedgehog signaling pathway
shRNA	Small hairpin RNA
siRNA	silencing RNA
STAT3	signal transducer and activator of transcription 3
TGFβ1	transforming growth factor beta 1
Tie2	angiopoietin receptor 2
TIMP-1	tissue inhibitor metalloproteinase 1
TKI	tyrosine kinase inhibitor
TNF-a	Tumor necrosis factor alpha
VEGFR2	Vascular endothelial growth factor receptor 2
